# Impact of P-Site tRNA and Antibiotics on Ribosome Mediated Protein Folding: Studies Using the *Escherichia coli* Ribosome

**DOI:** 10.1371/journal.pone.0101293

**Published:** 2014-07-07

**Authors:** Surojit Mondal, Bani Kumar Pathak, Sutapa Ray, Chandana Barat

**Affiliations:** 1 Department of Biotechnology, St. Xavier's College, Kolkata, West Bengal, India; 2 Dr. B.C Guha Centre for Genetic Engineering and Department of Biotechnology, Calcutta University, Kolkata, West Bengal, India; University of Lethbridge, Canada

## Abstract

**Background:**

The ribosome, which acts as a platform for mRNA encoded polypeptide synthesis, is also capable of assisting in folding of polypeptide chains. The peptidyl transferase center (PTC) that catalyzes peptide bond formation resides in the domain V of the 23S rRNA of the bacterial ribosome. Proper positioning of the 3′ –CCA ends of the A- and P-site tRNAs via specific interactions with the nucleotides of the PTC are crucial for peptidyl transferase activity. This RNA domain is also the center for ribosomal chaperoning activity. The unfolded polypeptide chains interact with the specific nucleotides of the PTC and are released in a folding competent form. *In vitro* transcribed RNA corresponding to this domain (bDV RNA) also displays chaperoning activity.

**Results:**

The present study explores the effects of tRNAs, antibiotics that are A- and P-site PTC substrate analogs (puromycin and blasticidin) and macrolide antibiotics (erythromycin and josamycin) on the chaperoning ability of the *E. coli* ribosome and bDV RNA. Our studies using mRNA programmed ribosomes show that a tRNA positioned at the P-site effectively inhibits the ribosome's chaperoning function. We also show that the antibiotic blasticidin (that mimics the interaction between 3′–CCA end of P/P-site tRNA with the PTC) is more effective in inhibiting ribosome and bDV RNA chaperoning ability than either puromycin or the macrolide antibiotics. Mutational studies of the bDV RNA could identify the nucleotides U2585 and G2252 (both of which interact with P-site tRNA) to be important for its chaperoning ability.

**Conclusion:**

Both protein synthesis and their proper folding are crucial for maintenance of a functional cellular proteome. The PTC of the ribosome is attributed with both these abilities. The silencing of the chaperoning ability of the ribosome in the presence of P-site bound tRNA might be a way to segregate these two important functions.

## Introduction

The synthesis of proteins and their folding into proper three dimensional structures is of fundamental importance for the maintenance of a functional cellular proteome. The ribosome is the cellular translation machine that can synthesize proteins using messenger RNA as the template and aminoacyl-transfer RNAs as substrates [1]. In the highly crowded cellular milieu a majority of synthesized large polypeptide chains require the assistance of a number of molecular chaperones to either fold or be rescued from misfolding and aggregation [2].

Several studies have demonstrated that the ribosome itself is capable of assisting in folding of proteins spanning a wide range of folds and functions [3]. The protein folding ability of the ribosome appears to be a universal one and has been demonstrated with ribosomes isolated from wide range of sources including eubacteria, archaebacteria, eukaryotes (yeast, rat liver and wheat germ), rabbit reticulocyte, bovine mitochondria and mitochondria of the parasite *Leishmenia donovani* [4]–[10]. The ribosome associated molecular chaperones like the complex of Hsp70 and J-type chaperones in the yeast *Saccharomyces cerevisiae* and trigger factor in *Escherichia coli* are reported to act as the first line of defense against protein aggregation of nascent translating polypeptide chains [11]. The chaperoning ability of the ribosome also includes it as one of the first chaperones to be encountered by newly synthesized proteins.

Surprisingly, despite of the large number of proteins associated with this ribonucleoprotein complex, like the peptidyl transferase ability, the chaperoning activity of the ribosome also originates in its ribosomal RNA. Both the peptidyl transferase activity and the chapernoning ability of the bacterial ribosome resides in the domainV of 23S rRNA of the bacterial large ribosomal subunit [3].The RNA corresponding to this domain, synthesized by *in vitro* transcription also has chaperoning ability and is referred to as bDV RNA in this study. Studies on the mechanisms of chaperoning activity of bDV RNA showed that it is a two step process involving its two sub-domains RNA1 and RNA2. The initial binding of the unfolded proteins take place with the RNA1 sub-domain (the central region of the PTC) followed by release of the proteins in their folding competent state by the RNA2 sub-domain (that consists of the remaining stem and loop structure of the RNA domain) [12]. Mutational studies were performed to identify nucleotides that are crucial for this protein folding function. The mutants that distort the central loop of RNA1 region, significantly affected the chaperoning ability and clustered mutations in the P-loop, markedly affected the activity of the RNA2 region of bDV RNA [13]. The protein folding activity has also been shown to be related to specific nucleotides in the large loop of the PTC since the same sets of nucleotide of the *E. coli* PTC interact with a diverse set of folding proteins [14]. In addition, studies have identified the ribosomal RNA as the target for two antiprion drugs 6-Aminophenanthridine (6AP) and Guanabenz (GA) in yeast *Saccharomyces cerevisiae* [5]. These studies demonstrated that the drugs inhibit the protein folding ability of the ribosome [5].

The mechanism by which the PTC executes its peptidyl transferase activity has been extensively studied. The ribosome has structurally distinct aminoacyl (A), peptidyl (P) and exit (E) sites that are rich in conserved RNA elements which stabilize tRNA and mRNA substrates in the structurally characterized ‘classical’(C) position via specific interactions. Classically bound L-shaped tRNA molecules orient perpendicular to the subunit interface such that their 3′-aminoacylated – CCA ends projects into the 50S subunit PTC, while the tRNA anticodon pair with mRNA codons within the small ribosomal subunit's decoding region. The 3′-CCA ends of the peptidyl- and aminocyl-tRNAs at the P-site and A-site are the acceptor and donor substrates of the peptidyl transferase reaction respectively. Their accurate positioning via interactions with specific nucleotides of the PTC is crucial for the peptidyl transferase reaction [15]. Studies with the antibiotics puromycin and blasticidin, that mimic the interactions between PTC and 3′- CCA ends of A- and P-site tRNAs respectively, have provided important insights into the mechanism of peptidyl transferase reaction [1], [16]. Hence, both the catalytic and chaperoning abilities of the ribosome depend on specific interactions between nucleotides of the PTC with either the 3′–CCA ends of the tRNAs or the unfolded protein [1], [14].The *in vitro* studies presented here explore the effect of tRNAs and antibiotics on the chaperoning ability of the *E. coli* ribosome and its bDV RNA using bovine carbonic anhydrase (BCAII) as the substrate protein. We demonstrate using mRNA bound 70S ribosomes (programmed ribosome) that a tRNA positioned at the P-site is more effective than an A-site tRNA in inhibiting ribosome's chaperoning function. We also show that the antibiotic blasticidin, a P-site substrate analog [1], is more effective in inhibiting the chaperoning ability of the ribosome and bDV RNA than either puromycin or the macrolide antibiotics (erythromycin and josamycin; [17]). Mutational studies of bDV RNA could identify the nucleotides U2585 and G2252 (both of which interact with P-site tRNA) to be important for bDV chaperoning ability. Taken together, the chaperoning ability of the ribosome is silenced in the presence of P-site bound tRNA, which might be a way to segregate the two important functions, peptidyl transferase activity and chaperoning ability, that reside in the same rRNA domain of the ribosome.

## Materials and Methods

### Preparation of ribosome and bDV RNA

Ribosomes were purified from *E. coli* MRE 600 cells [9] and its effect on refolding of the enzyme BCAII was performed as reported earlier [10]. The *in vitro* transcribed domain V of *E. coli* 23S rRNA (625 bp) is referred to as bDV RNA in this work. The DNA corresponding to this domain, cloned in plasmid pTZ57R/T, was a kind gift from laboratory of Professor C. Dasgupta (Univ. of Calcutta). The cloned DNA was linearized using EcoRI restriction enzyme (Fermentas) and the RNA was synthesized by run-off transcription using T7 RNA polymerase. The template DNA was removed with RNase free DNase (Fermentas), extracted using phenol-chloroform and the RNA was precipitated using ethanol.

### Protein denaturation and refolding studies

**Figure 1:**
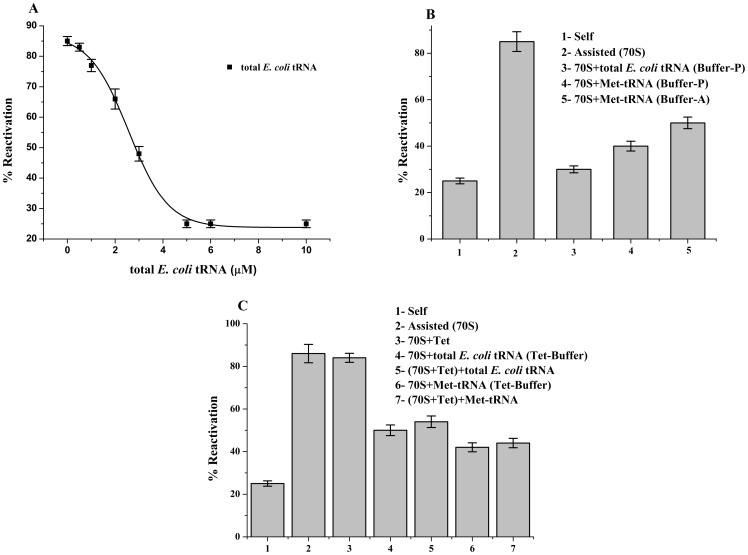
Effects of tRNA on chaperoning ability of non-programmed ribosome. (**A**) Dose dependent inhibition of ribosome's chaperoning ability due to binding of total *E. coli* tRNA. *E. coli* ribosome was allowed to bind to increasing concentrations of total *E. coli* tRNA in Buffer-P (Table S1). Reactivation of BCAII by the ribosome-tRNA complex was plotted (▪) against increasing total *E. coli* tRNA (▪) concentrations. The refolding experiments were repeated at least 3 times and the average values were plotted. The graph was fitted by Boltzmann fit. (**B**) Percent (%) reactivation of BCAII in presence of total *E. coli* tRNA and Met-tRNA bound to 70S ribosome in Buffer-A and Buffer-P (Table S1) are represented. (**C**) Effects of tetracycline on tRNA bound ribosome assisted refolding; Bar diagram represents a comparison of the BCAII reactivation (%) of; Self (1), 70S ribosome (2), 70S ribosome + tetracycline (3) 70S ribosome +5 µM total *E. coli* tRNA (4), 70S ribosome + tetracycline +5 µM total *E. coli* tRNA (5), 70S ribosome +5 µM deacylated Met-tRNA (6) and 70S ribosome + tetracycline +5 µM deacylated Met-tRNA (7). BCAII reactivation in absence of chaperone is marked as ‘Self’ in the figure.

**Figure 2:**
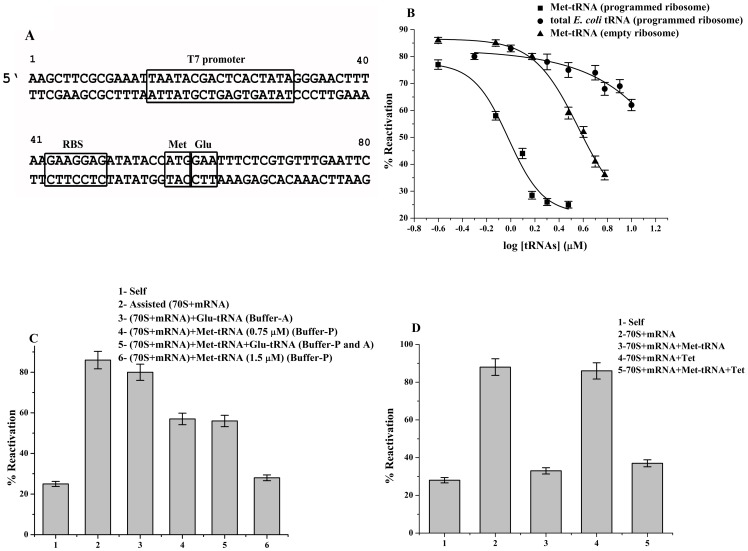
Effects of tRNA on programmed ribosome's chaperoning ability. (**A**) Schematic design of template for in vitro transcription of mRNA. The double stranded DNA was designed such that the ribosome binding site would position the methionine (AUG) and glutamic acid (GAA) codon at P-site and A-site respectively. These sequences are downstream of a T7 promoter sequence that was used for transcription of mRNA by T7 RNA polymerase (Materials and Methods). (**B**) Dose dependent inhibition of ribosome's chaperoning ability due to binding of tRNA. The *E. coli* ribosome programmed with mRNA was allowed to bind to increasing concentrations of Met- tRNA (▪) and total *E. coli* tRNA (•) in Buffer-P (Table S1). Inhibition of chaperoning activity of nonprogrammed ribosome (▴) in presence of increasing concentration of Met-tRNA is also plotted here. Reactivation (%) of BCAII by ribosome-tRNA complex was plotted against its increasing tRNA concentrations. The refolding experiments were repeated at least 3 times and the average values were plotted. The graph was fitted by Boltzmann fit. (**C**) Effect of presence of both deacylated Met-tRNA and Glu-tRNA on chaperoning ability of programmed ribosome. Bar diagram shows BCAII reactivation by the complexes 2 to 6; programmed ribosome (70S ribosome + mRNA) (2), Glu-tRNA bound programmed ribosome (70S ribosome + mRNA +1.5 µM Glu-tRNA) (3) and Met-tRNA bound programmed ribosome (70S ribosome + mRNA +0.75 µM Met-tRNA) (4). BCAII reactivation by complex 4 upon further addition of 0.75 µM of Glu-tRNA (5) or 70S ribosome + mRNA +1.5 µM Met-tRNA (6) is also shown. (**D**) Bar diagram represents a comparison of the BCAII reactivation (%) of; Self (1), 70S ribosome + mRNA (2), 70S ribosome + mRNA+1.5 µM Met-tRNA (3), 70S ribosome + mRNA + tetracyclin (4), 70S ribosome + mRNA+ tetracyclin +1.5 µM Met-tRNA (5).

**Figure 3:**
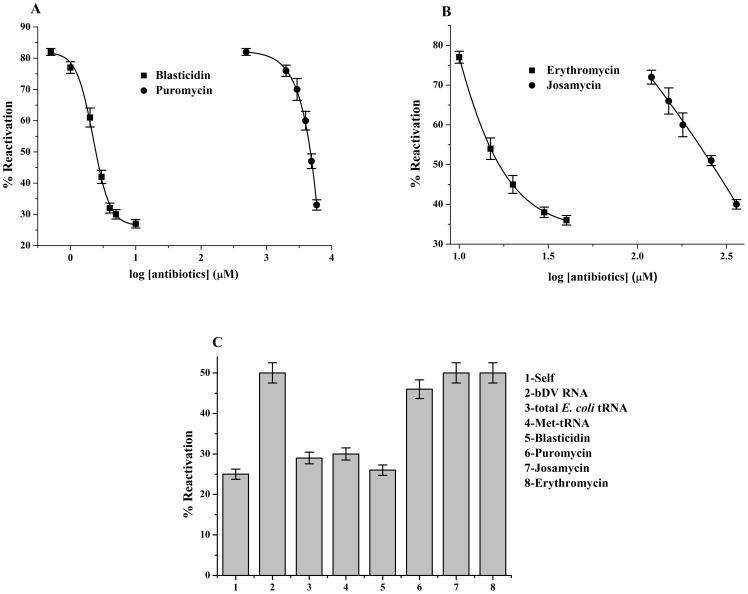
Effects of antibiotics on ribosome and domain V RNA assisted BCAII reactivation. (**A**) Dose dependent inhibition of ribosome's chaperoning ability due to binding antibiotics blasticidin and puromycin. *E. coli* ribosome was allowed to bind to increasing concentrations of the antibiotics in their respective buffers (Table S1). Reactivation of BCAII by ribosome-antibiotic complexes with antibiotics blasticidin (▪) and puromycin (•) are plotted against the log [antibiotics] (as indicated in x-axis of Fig. 3A). The refolding experiments were repeated at least 3 times and the average values were plotted. The graph was fitted by Boltzmann fit. (**B**) Dose dependent inhibition of ribosome's chaperoning ability due to binding of macrolide antibiotics. The ribosome was allowed to bind to erythromycin or josamycin in their respective buffers (Table S1). Reactivation of BCAII by ribosome-antibiotic complexes with antibiotics erythromycin (▪) and josamycin (•) bound ribosome were plotted against the log [antibiotics] (as indicated in x-axis of Fig. 3B).The refolding experiments were repeated at least 3 times and the average values were plotted. The graph was fitted by Boltzmann distribution fit. (**C**) A comparison of BCAII reactivation in presence of bDV RNA and bDV RNA complexed with total *E. coli* tRNA (15 µM), blasticidin (BLS; 3 mM), puromycin (PURO; 6 mM), erythromycin (ERY; 1.5 mM) and josamycin (JOSA; 2 mM) are shown in the bar diagram.

**Figure 4:**
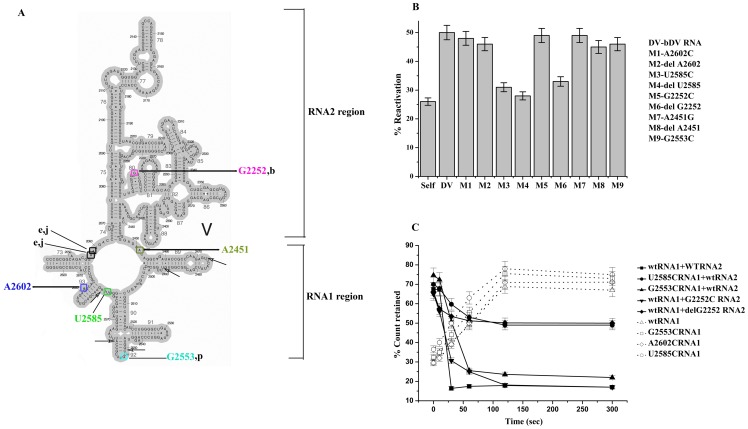
Effects of mutation on bDV RNA assisted BCAII reactivation. (**A**) The secondary structure of the bDV RNA has been shown here. The mutated nucleotides are also shown here using colour code. Nucleotides that are the binding sites of antibiotics blasticidin (b), puromycin (p), josamycin (j) and erythromycin (e) are also shown in the structure. The five specific nucleotides of bDV RNA which interacts with unfolded protein are also indicated by arrows. (**B**) Reactivation of BCAII in the presence of wild type (wt) and mutants *E. coli* bDV RNA. The reactivation of BCAII with mutated and wt bDV RNA was represented as bar diagrams. Mutated bDV RNAs are marked as M1 to M9 (M1- A2602C, M2- delA2602, M3- U2585C, M4- delU2585, M5- G2252C, M6- delG2252, M7- A2451G, M8- delA2451, M9- G2553C). BCAII reactivation in absence of bDV RNA is marked as ‘Self’ and in presence of wt bDV RNA is marked as ‘bDV RNA’. (**C**) Time course of binding of [α- ^32^P] labelled RNA1 to refolding BCAII. Unfolded BCAII was refolded in presence of [α- ^32^P] labelled wt RNA1 or mutant RNA1. Aliquots of samples were withdrawn at different time points from the RNA1-refolding protein mix and filtered through nitrocellulose. Percent (%) radioactivity retained (Materials and Methods) on the filter for wtRNA1 (Δ) and its mutants G2553C (□), A2602C (◊) and U2585C (○) are plotted against time. Time course of RNA2 mediated release of [α- ^32^P] labelled RNA1 from RNA1-BCAII complex. The [α- ^32^P] labelled RNA1 (wild type or mutated) was incubated with refolding BCAII for 5 min, to which RNA2 was added. Equal volume of samples were withdrawn at different time points and filtered through nitrocellulose. Percent (%) radioactive (materials and methods) retained on the filter for wtRNA1 + wtRNA2 (▪), wtRNA1 + G2252CRNA2 (▾), wtRNA1 + delG2252RNA2 (♦), U2585CRNA1 + wtRNA2 (•), G2553CRNA1 + wtRNA2 (▴) are plotted against time.

The denaturation and refolding of the BCAII protein either in presence or in absence of the ribosome or bDV RNA were performed under conditions reported in literature [10], [13]. Briefly, BCAII (Sigma Aldrich) (25 µM) was denatured to equilibrium with 6 M guanidine hydrochloride and 3.5 mM EDTA at 29°C for 3 hrs and refolding was initiated by 100-fold dilution in refolding buffer (Table S1) in presence of equimolar concentration of chaperone. The volume of the refolding mix was 300 µl. BCAII and the ribosome (or its complexes) were each present at concentration of 0.25 µM. The refolding mix was incubated at 29°C for a period of 30 minutes as reported earlier [10]. Recovery of enzymatic activity was assayed by adding 500 mM para-nitro-phenyl acetate (PNPA) to the refolding mixture and measuring the increase in absorbance of PNP at 420 nm with time [10]. Control experiments were performed in which BCAII was allowed to refold, in absence of any chaperone under the various buffer conditions stated below. The results obtained are referred to as ‘Self’ in this study (as shown in Fig. 1–4 and Fig. S1). The refolding of BCAII was unaffected under the conditions used in our studies.

The chaperone and its complexes used in this study are, a) the *E. coli* ribosome, b) the bDV RNA, c) ribosome–antibiotic complexes and d) ribosomes (mRNA programmed/non-programmed) complexed to total *E. coli* tRNA (Sigma Aldrich) or Met-tRNA or Glu-tRNA. As stated in Table S1, the formation of ribosomal complexes with the antibiotics and the tRNAs required appropriate buffer conditions. Care was taken to ensure that in each case control experiments of unassisted (self) folding and the ribosome assisted folding were performed under the same salt and buffer conditions. Control experiments were also performed to ensure that the tRNAs or antibiotics themselves do not affect self folding of BCAII under the conditions used in our study (Fig. S1). It is to be noted that *in vitro* studies on protein synthesis are carried out in buffers containing K^+^ as the positive cation. Replacement of NaCl with KCl in the refolding buffer did not affect reactivation yield both during assisted and unassisted refolding of BCAII.

### *In vitro* synthesis of mRNA

Double stranded oligonucleotides (synthesized from Sigma Aldrich) were designed (as outlined in Fig. 2A) and used as template for mRNA synthesis. The construct that had a T7 RNA polymerase promoter sequence followed by a ribosome binding site (Shine-Dalgarno sequence) upstream of the codons ATG and GAA respectively. An mRNA fragment containing a Shine-Dalgarno sequence has been reported to bind tightly to 70S ribosome [18]. This would ensure that the mRNA oligomer does not dissociate from the 70S ribosome under the conditions of the experiment. This sequence would also appropriately position the Met-tRNA and Glu-tRNA in the P- and the A-site of the mRNA programmed ribosome [19], [20]. The DNA strands were annealed and used as template for *in vitro* transcription following which the template DNA was digested with RNase free DNase. The RNA was precipitated with ethanol, centrifuged and resuspended in DEPC treated water and the concentration of mRNA determined by measurement of optical density at 260 nm.

### Deacylated tRNAs

It has been reported that in solutions of near-neutral pH [9], [10], the aminoacyl-tRNAs spontaneously deacylate with half-lives that range from minutes to tens of minutes [21]. Hence, under our experimental conditions as stated above (pH of refolding buffer is 7.5 and BCAII activity is measured after 30 minutes of refolding), much of the aminoacylated tRNAs are likely to be deacylated. Therefore in our present study deacylated Met-tRNA and Glu-tRNA have been used. Deacylated Met-tRNA was purchased from M.P. Biomedicals and deacylated Glu-tRNA was prepared as described below. *E. coli* Glu-tRNA was isolated from the overproducing plasmid pKR15 in *E. coli* DH5α strain (kindly gifted by Pr J. Lapointe, University of Laval, Quebec, Canada) and the growth condition and the purification procedure were followed [22]. *E. coli* DH5α containing the overproducing plasmid pKR15 was grown at 37°C under aerobic conditions in M9 minimal medium with 11 mM dextrose and 15 mM thiamine, harvested at the end of the exponential phase and resuspended in 20 mM Tris-HCl, pH 7.2. RNA was extracted with phenol, precipitated from the aqueous phase with 2 vol. of ethanol and 0.1 vol. of potassium acetate 20% pH 6.0, and dissolved in 1M NaCl. After centrifugation at 8000 g during 40 min, the clear RNA solution was diluted fivefold with water and loaded on a column of Q Sepharose (Pharmacia) which was then washed with 200 mM NaCl (in 50 mM Tris-HCl, pH 7.2) before eluting the tRNA with 1.5 M NaCl (in 50 mM Tris-HCl, pH 7.2). This unfractionated tRNA was deacylated by incubation at 37°C for 20 minutes in 0.5 M Tris-HCl, pH 8.9. Purification of the glutamate accepting tRNAs from 1000 A_260_ units of tRNA was performed by chromatography on a 21.2 mm×250 mm column of C18 ODS-Hypersyl (Shandon) coated with trioctylammonium chloride, using a linear salt gradient made with 0.5 M CH3COONH4 pH 4.5, 0.2 mM dithiothreitol, 1 mM EDTA and 5 M CH3COONH4 pH 6.0, 0.2 mM dithiothreitol, 1 mM EDTA. Each glutamate accepting tRNA was purified to homogeneity by 2 or 3 successive passages on this column (until the peaks were symmetrical). Samples were concentrated with a Q-Sepharose FF column (Pharmacia) and desalted using Centricon-10 devices (Amicon). Purified Glu-tRNA was quantified and the accepting capacity of Glu-tRNA was measured [22]. The glutamate accepting capacity of the purified Glu-tRNA was found to be 1100 pmol per *A*_260_ units.

### *In vitro* binding of tRNA and antibiotics with ribosome and bDV RNA

#### Binding of tRNA to the empty ribosome

The binding of total *E. coli* tRNA, deacylated Met-tRNA or Glu-tRNA to the ribosome were performed in Buffer-P or Buffer-A [23]. The conditions of binding and the composition of the buffers that facilitated binding of tRNA to the P-site and A-site respectively were obtained from literature and are stated in Table S1. Total *E. coli* tRNA bound empty ribosomes were prepared by incubating 70S ribosome (0.25 µM) with total *E. coli* tRNA (0.5 µM – 10 µM; as indicated in x-axis of Fig. 1A) in 297 µl of Buffer-P for 1 hr at 37°C. Met-tRNA bound empty ribosomes complexes were prepared under similar conditions in presence of 5 µM Met-tRNA in either Buffer-P or Buffer-A. Denatured BCAII (3 µl) prepared as described above was added to the ribosome-tRNA complexes, incubated at 29°C for 30 minutes and BCAII reactivation yields were determined as stated above. The concentration of the chaperone (70S-tRNA) in refolding mix is 0.25 µM which is at equimolar ratio with that of the refolding protein.

To study the effect of blocked A-site of empty ribosome on tRNA mediated inhibition of chaperoning ability, the ribosome was first allowed to bind to tetracycline and the effect of total *E. coli* tRNA or Met-tRNA on the chaperoning ability of the ribosome was measured. For tetracycline binding to ribosome, ribosome (0.25 µM) was incubated with 3.75 mM tetracycline, total *E. coli* tRNA (5 µM) or Met-tRNA (5 µM) in 297 µl of Tet-Buffer [24], (Table S1).The ability of these complexes to increase BCAII reactivation was measured. The effect of percent (%) BCAII reactivation of tetracycline bound ribosome was also studied. Control experiments with tetracycline showed that the antibiotic did not influence the self folding of BCAII under the conditions used in our study.

#### Binding of tRNA to the programmed ribosome

The *in vitro* transcribed mRNA, prepared as described above bound to the 70S ribosome is referred to as programmed ribosome in this study. The binding of mRNA and Met-tRNA (P-site) or Glu-tRNA (A-site) to the ribosome were performed in Buffer-P and Buffer-A respectively (Table S1). Met-tRNA bound programmed ribosomes were prepared by incubating 70S ribosome (0.25 µM) with 2 fold molar excess of mRNA and Met-tRNA (0.25 µM-3.0 µM; as indicated in x-axis of Fig. 2B) in 297 µl of Buffer-P for 1 hr at 37°C [24]. Experiments with the empty ribosome (in absence of mRNA) and increasing concentrations of Met-tRNA were also performed under similar conditions to assess the effect of mRNA directed positioning of the tRNA at the P-site. Similar experiments were also conducted to study the effect of increasing concentrations of total *E. coli* tRNA on chaperoning ability of programmed ribosomes. The effect of tetracycline on chaperoning ability of programmed ribosome and Met-tRNA bound programmed ribosome were also studied using conditions as stated above. Glu-tRNA bound programmed ribosomes were prepared by incubating 70S ribosome (0.25 µM) with 2 fold molar excess of mRNA and Glu-tRNA (1.5 µM; as indicated in x-axis of Fig. 2B) in 297 µl of Buffer-A for 1 hr at 37°C. The effect of these complexes in BCAII reactivation was measured. Control experiments were also performed to confirm that the mRNA and tRNAs do not influence the self folding of BCAII under the conditions used in our study (Fig. S1A).

#### Binding of antibiotics to the empty ribosome

Ribosomes-antibiotic complexes were prepared by incubating 0.25 µM ribosome with increasing concentrations of each antibiotic in 297 µl of their respective binding buffers (Table S1) at 37°C for 20 minutes and then at 20°C for 15 minutes. Specific conditions reported in literature, unique to binding of each of the antibiotics (Sigma Aldrich) were used to ensure binding [25]–[27]. Denatured BCAII (3 µl) was added to the ribosome-antibiotic complex, incubated at 29°C for 30 minutes and BCAII reactivation yields were determined. The final concentration of both BCAII and the chaperone (ribosome-antibiotic complex) in the refolding mix was 0.25 µM. Care was taken to ensure that unassisted (self) and the ribosome assisted folding were also performed under the same salt and buffer conditions. Control experiments were also performed to ensure that the antibiotics themselves do not affect self folding of BCAII under the conditions used in our study (Fig. S1A).

#### Binding of antibiotics and tRNA to bDV RNA

As stated earlier, the *in vitro* transcribed RNA (corresponding to domain V of 23S rRNA) can also assist in protein folding albeit to a lower extent than that compared to the ribosome [12]. Hence, most of the analysis of the mechanism of ribosome's chaperoning function that has been reported in literature has been performed on the *in vitro* transcribed bDV RNA [12], [13], [28]. The effects of tRNA and antibiotics on the chaperoning activity of this RNA domain were therefore studied using denatured BCAII as the substrate protein. For refolding studies the RNA was allowed to bind to the total *E. coli* tRNA, Met-tRNA and antibiotics using the specific buffer conditions necessary for their binding (Table S1). Care was taken to ensure that control (self) and the bDV RNA (assisted) folding were also performed under the same salt and buffer conditions.

### Preparation of wild type and mutant segments of bDV RNA

The segments corresponding to RNA1 and RNA2 fragments of bDV RNA were cloned in the pTZ57R/T vector downstream of the T7 RNA polymerase promoter. While the sequence of RNA2 is consecutive in the domain, the RNA1 region had to be generated by joining two discontinuous segments RNA1a (27 nucleotide fragment corresponding to 5′ end of RNA1) and RNA1b (185 nucleotide fragment corresponding to the 3′ half of RNA1) using XbaI (Fermentas) restriction site. Mutations that were introduced in DNA corresponding to bDV RNA, RNA1 and RNA2 were confirmed by sequencing. The RNA was synthesized by run-off transcription as described earlier.

### Site directed Mutagenesis

Mutations U2585C (in the RNA1 region) and delG2252 (in the RNA2 region) were available in the laboratory. Mutations delU2585, G2252C, A2602C, delA2602, A2451G, delA2451, G2553C were introduced in appropriate positions of DNA corresponding to bDV RNA, bDV RNA1 and bDV RNA2. These mutants were generated using appropriate primers and site directed mutagenesis kit (Stratagene). The mutations were confirmed by sequencing.

### Filter binding assay

Filter binding studies were performed to compare binding and release of the refolding protein with wild type (wt) and mutant RNA1 and RNA2 using the procedure as reported earlier [13]. Briefly wt or mutated [α- ^32^P] labelled RNA1 were incubated with equimolar concentration of refolding BCAII for different time intervals, filtered through 0.22 µM pre-soaked nitrocellulose filter paper (Millipore), and ^32^P counts retained on the filter measured by liquid scintillation counter (Perkin Elmer). Comparing the radioactive count incorporated in the total RNA to that on the filter, the percentage of radioactivity retained was calculated. To follow the RNA2 mediated release of RNA1 from the RNA1-protein complex, the unfolded protein was allowed to form a stable complex with the [α- ^32^P] labelled wt RNA1 for 5 minutes. Mutant or wt unlabeled RNA2 was then added to the reaction mixture at the same concentration as RNA1. Aliquots withdrawn at different time intervals were filtered through nitrocellulose filter paper and radioactivity retained on filter paper measured. In all these studies the wt counterpart of RNA1 and RNA2 served as the control. In filter binding studies with bDV RNA or its mutants, the radiolabelled RNA was incubated with refolding BCAII. The samples were withdrawn at different time intervals, cross-linked at 254 nm for 90 seconds (GS Genelinker, Biorad), filtered through nitrocellulose filter paper and the radioactivity retained on the filter were determined.

### Circular dichroism Spectroscopy

Circular dichroism spectroscopy was performed with bDV RNA and its mutants (M1-M9). For CD measurements the *in vitro* transcribed RNAs (0.1 µM) were dissolved in 20 mM sodium phosphate buffer (pH 7.6) and the CD spectra were recorded from 200 to 300 nm using 1 cm quartz cuvette in a Jasco J-815 CD spectrometer.

## Results

### Effect of tRNA on ribosome assisted chaperoning activity

#### Studies with non-programmed *E. coli* 70S ribosome

Establishment of proper interactions between the PTC and its two substrates, the 3′-CCA ends of A- and P-site tRNAs, are crucial for peptidyl transferase activity of the ribosome [1]. An earlier studies using *in vitro* transcribed tRNA bound to bDV RNA under buffer conditions conducive to P-site binding had indicated that the P-site tRNA inhibits the protein folding ability of this RNA domain (stated as unpublished observation [3]). In order to investigate the effect of total *E. coli* tRNA, we allowed it's binding to the *E. coli* 70S ribosome in Buffer-P (Table S1) and the effect of ribosome-tRNA complex on refolding of denatured BCAII was studied. As shown in Fig. 1A, a dose dependent inhibition of BCAII reactivation was observed with increasing tRNA concentrations present during formation of the ribosome-tRNA complex. Total *E. coli* tRNA at a concentration of 5 µM was capable of complete inhibition of ribosome's chaperoning activity (BCAII reactivation observed was 30% that is comparable to self folding; Fig. 1B). The affinity of P-site tRNA to non-programmed ribosome is reported in literature to be less than 10^5^ M^−1^ at 10 mM Mg^2+^ [29]. As shown in Fig. S1A, deacylated Met-tRNA has no effect on BCAII self folding. Since the tRNAs are polyanions, control experiments on ribosome's chaperoning ability in presence of another polyanion, heparin, were also performed under the conditions stated above. The chaperoning function of the ribosome showed no inhibition in presence of heparin (Fig. S1B) further confirming the specificity of the observed tRNA mediated inhibition.

Deacylated Met-tRNA was then used to study the effect of a specific deacylated tRNA on ribosome mediated folding. Since tRNA positions on the ribosome are highly sensitive to buffer conditions [30] the effect of the tRNA on ribosome's chaperoning ability was tested in Buffer-P and Buffer-A (Table S1). As shown in Fig. 1B, ribosome bound to Met-tRNA (5 µM) showed 40% and 50% BCAII reactivation in Buffer-P and Buffer-A respectively. Since inhibition of chaperoning activity was observed in both Buffer-A and Buffer-P, which of the two tRNA binding sites is responsible for inhibition needed to be deciphered. For this purpose, the A-site of the ribosome was blocked by tetracycline under its appropriate binding conditions (Table S1). Tetracycline is an antibiotic that binds to the A-site of the 30S subunit and prevents tRNA binding to the A-site of the ribosome [31]. The effect of total *E. coli* tRNA (5 µM) and Met-tRNA (5 µM) on folding ability of tetracycline bound ribosome was then tested. Appropriate control experiments of tRNA mediated inhibition of ribosome's chaperoning ability in the same buffer were also performed. The inhibition observed in presence of both tRNAs remains essentially unchanged (Fig. 1C) even when the A-site has been blocked with tetracycline. These evidences indicate that binding of P-site tRNA to ribosome is responsible for inhibiting ribosome's chaperoning function.

#### Studies with programmed E. coli 70S ribosome

For appropriate positioning of the tRNAs, further studies were performed with mRNA programmed ribosomes in which the tRNAs would be directed to bind to the A- and the P-site of ribosome based on codon-anticodon interaction. The mRNA sequence had the AUG (Met) and GAA (Glu) codons, 7 bp downstream to a ribosome binding site (Shine-Dalgarno sequence). Since, the Shine-Dalgarno (SD) sequence increases the affinity for mRNA binding to the ribosome by an order of magnitude [19]; this sequence would ensure tight binding of the mRNA to the ribosome. The presence of mRNA would also increase the specificity of selection of P-site tRNA as reported earlier [21]. In addition, the SD sequence has also been shown to play a role in increasing the specificity of binding of cognate tRNA at the codon following the first AUG [20]. Thus programming of the ribosome with the mRNA sequence as shown in Fig. 2A would ensure the positioning of deacylated Met-tRNA and Glu-tRNA on the AUG and the GAA codons of the ribosome's P-site and A-site respectively. The mRNA was synthesized as described in Materials and methods section. The ribosome programmed with the mRNA could assist in BCAII reactvation (Fig. 2C). Programmed ribosomes were allowed to bind to increasing concentrations of deacylated Met-tRNA (Materials and Method) and the ability of this complex to assist in reactivation of BCAII was tested. As shown in Fig. 2B, a dose dependent inhibition of chaperoning ability was observed with complete inhibition attained in presence of 1.5 µM of P-site specific Met-tRNA. Binding of Glu-tRNA to A-site of programmed ribosome showed only marginal inhibition even at concentrations in which Met-tRNA showed significant inhibition ([Glu-tRNA]  = 1.5 µM) (Fig. 2C).

In the absence of mRNA, comparable Met-tRNA mediated inhibition of ribosome's chaperoning ability was achieved only at a concentrations approximately four fold higher (Fig. 2B) than that observed with the programmed ribosome. This testifies for the mRNA mediated positioning of the P-site tRNA. Fig. 2B also shows that unlike the empty ribosome, only partial inhibition of the chaperoning ability of the programmed ribosome was achieved even at high concentrations (10 µM) of total *E. coli* tRNA. This might be expected only if the mRNA programming of the ribosome had led to increased specificity of the P-site for Met-tRNA, which represents only a subpopulation of the total *E. coli* tRNA. Earlier studies have also demonstrated that while in absence of mRNA, the P-site tRNA establishes contacts almost exclusively with the 50S subunit, additional contacts between the tRNA and the 30S subunit is established upon codon-anticodon interaction in presence of mRNA [21]. These studies also showed that in presence of mRNA the specificity of selection of P-site tRNA is also significantly increased.

Next, the ribosome was bound to Met-tRNA at a concentration at which partial inhibition of the ribosome's chaperoning ability was observed (Fig. 2A) ([Met-tRNA]  = 0.75 µM). Upon increasing the concentration of Met tRNA (P-site) further inhibition was observed while Glu-tRNA was completely ineffective (Fig. 2C).

The effect of Met-tRNA on tetracycline bound programmed ribosome was also studied. The A-site of the programmed ribosome was blocked by tetracycline under its appropriate binding conditions [24]. Presence of Met-tRNA (1.5 µM) caused comparable inhibition of chaperoning ability of programmed ribosome in presence or absence of tetracycline thus indicating at the importance of P-site tRNA in inhibition of ribosome's chaperoning function. Control experiments were also performed to verify that the mRNA, tRNAs or the antibiotic tetracycline does not affect the self refolding of BCAII under the conditions used in our study.

Together these studies imply that the tRNA positioned at the P-site of the ribosome and not its A-site is effective in inhibiting ribosome's chaperoning activity. As stated above, these observations were made with deacylated tRNAs since an aminoacylated tRNA is likely to be deacylated under the conditions used in our study. It is to be noted that some studies suggest that there is no difference in binding affinity of acylated vs. deacylated tRNA for the P-site of the ribosome [32], [33]. Biochemical experiments however show that while N-acetyl-aminoacyl-tRNA occupies P-site of both subunits, i.e. P/P site, deacylated tRNA can spontaneously fluctuate between the P/P and P/E states [34]. Hence the inhibition of the chaperoning action as stated above could be due to the P-site tRNA residing in either of these states.

### Effect of antibiotics on ribosome's chaperoning activity

#### Studies with antibiotics that are PTC substrate analogs

The antibiotics blasticidin and puromycin act as PTC substrate analogs and their interaction with 23S rRNA mimic those between the PTC and the 3′ –CCA ends of the P- and A- site tRNAs respectively (Fig. 4A). The antibiotic blasticidin acts by interacting with G2252 or G2253 of the PTC P-loop [1]. Crystal structure of the complex of *H. marismortui* 50S subunit with CCdAp-puromycin, shows the puromycin moiety to be positioned at the A-site and interacting with nucleotide G2553 of the A-loop [16]. To confirm whether the observed inhibition (Fig. 1 and Fig. 2) of ribosome's chaperoning ability was due to the interaction between the domain V of 23S rRNA and the 3′–CCA end of P-site tRNA or A-site tRNA, the effect of these antibiotics on ribosome assisted protein folding was tested.

The antibiotics were allowed to bind to the ribosome under optimum conditions reported in literature (Table S1). As shown in Fig. 3A and Fig. 3B, a dose dependent decrease of chaperoning ability (reflected in % reactivation of BCAII) was observed with increasing concentration of antibiotics. As shown in Fig. 3A, complete inhibition of ribosome's chaperoning activity was observed at a concentration of 5 µM of blasticidin, that is of the same order of magnitude as the K_d_ for the antibiotic [35] (Table S2). This study confirmed that the inhibition of chaperoning activity observed with P-site tRNA (shown above) was indeed due to the interaction between the P-loop of PTC and the 3′–CCA end of tRNA positioned in the P/P state.

However, even minimal inhibition of ribosomal chaperoning ability was observed only at a high concentration of puromycin (greater than 5 mM) (Fig. 3A). Puromycin has been reported to bind very weakly to empty ribosomes, has a low binding constant for *E. coli* ribosomes [36] and nucleotides of the 23S rRNA shows altered chemical reactivities in the presence of puromycin only at high antibiotic concentrations. This might be the reason why high concentration of puromycin is needed to achieve complete inhibition of ribosomal chaperoning activity. Further, puromycin being an analog of the donor substrate, might bind weakly in the P- site of empty ribosome. The inhibition observed at the high concentration of the antibiotic in this study might also be due to its residual binding to the P-site.

#### Studies with macrolide antibiotics

The macrolide antibiotics block the polypeptide exit tunnel of the ribosome and thus inhibit extrusion of the nascent chain [17]. They bind to the exit tunnel hydrophobic crevice at the PTC and also establish contact with nucleotides of domain V of the 23S rRNA. Erythromycin and josamycin are 14- and 16-membered macrolides respectively and the longer disaccharide extension at position C5 of its macrolactone ring allows josamycin to approach the PTC more closely than other 14- and 15-membered macrolides. The nucleotides of the PTC that interact with erythromycin and josamycin are shown in Fig. 4A.

Our studies with erythromycin and josamycin show that both these antibiotics can inhibit ribosome mediated protein folding in a dose dependent manner with maximal inhibition achieved at concentrations much higher than their K_d_ [37] as indicated in Fig. 3B and Table S2. (Erythromycin: maximal inhibition obtained at 40 µM; Josamycin: maximal inhibition obtained at 360 µM).

### Effect of tRNA and antibiotics on bDV RNA chaperoning ability

As stated earlier, the *in vitro* transcribed RNA corresponding to domain V of 23S rRNA (bDV RNA) can also assist in protein folding. Studies using this RNA have provided several important insights into the mechanism of the ribosome's chaperoning activity [12], [13]. Studies on the effect of total *E. coli* tRNA, deacylated Met-tRNA and the antibiotics on bDV RNA mediated chaperoning activity were therefore performed. While total *E. coli* tRNA, deacylated Met-tRNA, blasticidin showed complete inhibition at concentrations indicated in legend of Fig. 3C, puromycin showed only marginal effect on bDV RNA mediated chaperoning action. This further emphasizes the importance of interaction between PTC nucleotides and the 3′–CCA end of P-site tRNA in determining the tRNA mediated inhibition stated above.

Studies on the effect of macrolide antibiotics on domain V RNA mediated chaperoning ability were performed. Neither erythromycin nor josamycin could inhibit the chaperoning activity (Fig. 3C). The macrolide antibiotics bind above the constriction formed by the proteins L4 and L22 at the entry of the polypeptide tunnel [38]. Mutations in these ribosomal proteins cause macrolide resistance [39] indicating their importance in binding of macrolide antibiotics. Hence, the lack of inhibition of chaperoning ability of bDV RNA by macrolides is expected since these antibiotics are unlikely to bind to *in vitro* transcribed RNA. This also provides additional support to the fact that the observed inhibition of the chaperoning action with blasticidin or puromycin observed here is not due to any nonspecific effect.

Taken together, the studies with blasticidin (P-site analog) and puromycin (A-site analog) further establish that the interactions between 3′–CCA end of the tRNA and P-loop and not the A-loop are inhibitory for the chaperoning action of bDV RNA and the ribosome. In this study we also show that the macrolides that target the exit tunnel hydrophobic crevice at the PTC affect chaperoning ability of the ribosome but not of its bDV RNA.

### Effect of mutations on bDV RNA chaperoning ability

#### Refolding ability of bDV RNA mutants

Based on the studies stated above, we introduced mutations in bDV RNA at sites essential for P-site tRNA binding. Universally conserved nucleotides like G2252, U2585 and A2451 have been shown to be involved in P-site tRNA binding [40]. The A2451 nucleotide has also been proposed to play a major role in catalysing the peptide bond formation [1]. We also targeted conserved nucleotides G2553 (essential for A-site binding of tRNA) and A2602 (conformationally the most flexible nucleotide of the PTC, the orientation of which varies depending upon the nature of the bound PTC substrate [41]. Mutations were introduced in DNA corresponding to bDV RNA and the mutant RNAs were synthesized by *in vitro* transcription as stated in ‘Materials and Methods’. Fig. 4A shows the secondary structure of bDV RNA in which the positions of the mutations have been indicated. Circular dichroism spectra of the wild type and the mutant RNAs (Fig. S2A) confirmed that the mutations did not lead to any global change in structure of the bDV RNA.

A comparison of the effect of deletion or substitution mutations at these sites on chaperoning ability of bDV RNA is shown in Fig. 4B. Substitution mutation at 2585 (U2585C (M3)) leads to loss of chaperoning ability of bDV RNA. While a mutation at G2252 (G2252C (M5)) did not show any effect, the deletion of the nucleotide (delG2252 (M6)) led to complete loss of chaperoning ability of bDV RNA. This might imply a role of 2′-OH of this nucleotide since the 2′-OH of ribose moieties of specific domain V nucleotides have also been proposed to play significant role in other PTC related functions [42], [43]. However, deletion of G2252 (M6) might also have lead to a subtle change in the conformation of this domain thus accounting for its loss of activity. It is to be noted that this nucleotide is also crucial for binding of the antibiotic blasticidin with which we observed complete inhibition of chaperoning ability of both the ribosome and bDV RNA (Fig. 3). Neither A2451G (M7) mutation (a substitution that leads to loss of peptidyl transferase activity of the ribosome; [44]) nor its deletion (M8) significantly affected domain V chaperoning ability. Similarly, with the G2553C (M9) mutation (in the A-loop) the folding ability of bDV RNA remains essentially unaltered. No effect on refolding yield was observed with either mutation or deletion of A2602 (M1 and M2).

Hence, of the nine point mutations studied here, two mutations resulted in a significant reduction in chaperoning activity of bDV RNA. In this context, recently reported studies on refolding ability of mutated variants of domain V of 23S rRNA requires mention [45]. Each of these mutants has multiple mutations in selected regions of the RNA domain. These mutations did not lead to any global change in the structure of bDV RNA and it was confirmed that the *in vitro* transcribed domain V rRNAs are structured under the refolding condition. However, most of these mutants showed highly reduced refolding activity, less than 50% compared to the wild type. Mutations in regions corresponding to the protein or 6AP (stated above) interaction sites (UUG2492-94, UU2561-62, UAG2586-88 on 23S rRNA) showed highest defect in their chaperoning activity. Of the two mutants of bDV RNA that had significantly reduced chaperoning ability in our study, the U2585C mutation lie in proximity to one of the aforementioned RNA-protein interaction sites.

#### Binding and release of refolding protein by RNA1 and RNA2 mutants

As stated earlier the bDV RNA mediated chaperoning is a two step process in which the RNA1 sub-domain initially binds the refolding protein and the RNA2 sub-domain releases the bound protein in a folding competent state. As reported earlier, filter binding studies with wild type or mutant RNA1 and RNA2 have provided insights into the underlying mechanism of loss of chaperoning activity of the bDV RNA mutants. Mutations were introduced in the DNA corresponding to RNA1 (G2553C, A2602C, U2585C) and RNA2 (G2252C, delG2252) as described in “Materials and Methods”. In the filter binding assay, the [α- ^32^P] labelled RNA would be retained on the filter paper only if bound to the refolding protein, while the unbound RNA passes through it. The time course of interaction between RNA1 (mutant or wild type) and the refolding protein and its subsequent RNA2 (mutant or wild type) mediated release would therefore be reflected by the amount of radioactivity retained on the filter.

As shown in Fig. 4C, a comparable time course of binding with wt RNA1 and its mutants (G2553C, A2602C, U2585C) (dashed line), indicates that none of these mutations affect the overall binding interaction between bDV RNA and the refolding protein. A comparison of the ability of wt or mutated RNA2 (G2252C, delG2252) to mediate time dependent release of RNA1 bound protein is also shown in Fig. 4C (solid line). The RNA2 mutant, RNA2delG2252, was capable of mediating only partial release of the wtRNA1 bound protein. The protein bound to the RNA1 mutant RNA1U2585C also could not be released in presence of wtRNA1. Control experiments in which native BCAII was allowed to interact with radiolabelled bDV RNA or RNA1, no radioactivity was retained on the filter signifying lack of interaction between the chaperone and the the properly folded protein.

Parallel ongoing experiments in our laboratory on the effect of bDV RNA on aggregation and refolding of molten globule form of BCAII had also shown that the delG2252 and U2585C mutant could efficiently suppress BCAII aggregation due to their inability to release the bound protein (Pathak B.K. et al (2014), PLoS One. 9(5): e96425). In filter binding studies with wt bDV RNA and its mutant bDV RNAdelG2252, a lack of release of the protein is observed with the mutant RNA (Fig. S2B). While BCAII bound to bDV RNAdelG2252 mutant does not show increase in reactivation over that of self folding, addition of wtRNA2 enables release and reactivation of the bound protein (Fig. S2C). This confirms that inability to release the bound protein is indeed the cause of loss in chaperoning activity of the mutant. Taken together, these studies indicate at a role of both the nucleotides U2585 and G2252 in the release process of the refolding protein. However further studies with mutant ribosomes are necessary to confirm these observations.

## Discussion

**Figure 5:**
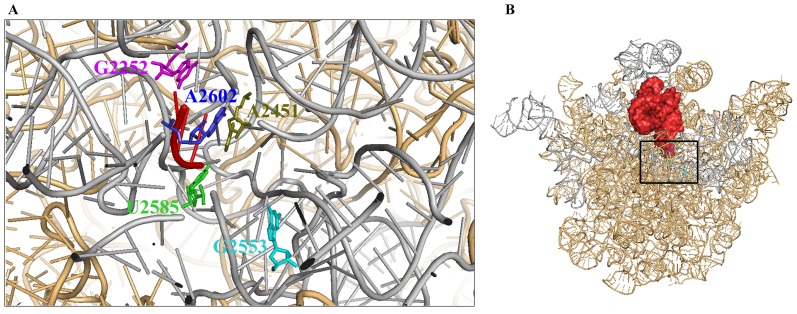
Representation of the structure of *E. coli* ribosome with P-site tRNA. (**A**) The 23S rRNA of *E. coli* large ribosomal subunit (PDB: 2I2V) has been displayed (light orange) here with 3′-CCA end of P-site tRNA (PDB: 2J02). The domain V region of 23S rRNA has been shown in grey and the 3′-CCA end of P-site tRNA is represented in red. The nucleotides which have been mutated in this study have also been shown in sticks and are represented in the same colour as in Fig. 4A. (**B**) The 23S rRNA of *E. coli* large ribosomal subunit (PDB: 2I2V) has also been displayed (light orange) here with P-site tRNA (PDB: 2J02) (red colour surface representation). This view is in the same orientation in which Fig. 5A has been shown.

The role of the ribosome as the cellular protein synthesis machine is well established. The ability of the ribosome to assist in refolding of proteins [3] without the participation of any co-chaperone or cofactor (e.g. ATP) has also been extensively studied. Both these activities i.e. synthesis and folding of proteins are central to the maintenance of a functional cellular proteome. However, since the ribosome is the only translational machine of the cell, its translational ability is critical for cell viability. Our studies demonstrate that the binding of P-site tRNA to empty or programmed ribosome leads to inhibition of ribosome's chaperoning ability. Studies using antibiotics that act as PTC substrate analogs indicated that the interaction between 3′-CCA end of P-site tRNA with domain V of 23S rRNA of the ribosome (Fig. 5) is responsible for the observed inhibition. Ribosomal ligands (tRNAs or antibiotics), targeted to bind to specific sites on the PTC were used in this study. A comparison of the relative doses of these ligands to titrate out ribosome's chaperoning ability, in presence of appropriate internal controls, form the basis of the conclusions presented here. This study relies on the optimum conditions for binding of tRNAs or antibiotics to the ribosome as reported in literature. Further studies are necessary to determine the exact occupancy of the ribosome with its substrates under our experimental conditions and to correlate the inhibition of ribosome's chaperoning function with the concentration of the ribosome substrate complex. Mutagenesis studies presented here have identified two nucleotides that are necessary for tRNA binding [40], U2585 and G2252 to be important for chaperoning ability of bdV RNA. Both these mutants showed a deficiency in the release of the bound protein, which is an important step in the mechanism of bDV RNA mediated protein folding. Thus, whether the presence of a P-site tRNA prevents access of the refolding protein to the domain V of 23S rRNA of the ribosome or interferes with the mechanism of the ribosome mediated refolding process also needs to be further ascertained.

The discussion of some recent observations are relevant in context of the above studies that imply that actively translating ribosomes with a tRNA positioned at the P-site would be unable to perform their chaperoning function. Firstly, the question as to whether the ribosome acts as a chaperone by trans (that is on other proteins) or cis (that is on the same polypeptide that it has synthesized) mechanism still remains to be answered. In the cis-mechanism, the conflict of the two concepts, the ribosome exit tunnel and protein folding on the PTC of the 50S subunit arises where indeed the question of how the nascent protein associates with the PTC after being released from the exit site of the tunnel needs to be further resolved [3]. In this perspective, whether the chaperoning ability of the translating ribosome is activated after release factor mediated termination of polypeptide synthesis i.e. when the deacylated P-site tRNA moves to the P/E state, requires further investigation. Secondly, recent *in vivo* studies using PSI^+^ and Ure 3 strains of yeast *Saccharomyces cerevisiae* have identified the ribosomal RNA as target for two antiprion drugs, 6AP and GA [5]. It was also demonstrated that these compounds selectively inhibit the protein folding ability of the ribosome and that the competitive obstruction of the protein binding sites of 23S rRNA by 6AP forms the basis of the inhibitory effect of the drug [45]. These studies imply that the protein folding ability of the ribosome might also have impact on diverse cellular activities. Thirdly, our studies also raise the possibility that the ribosomes not engaged in active translation or its isolated 50S subunit present in the cell would be involved in assisting cellular protein folding. This is further plausible since the chaperoning ability of isolated 50S subunit has been shown to be greater than the 70S ribosome [46]. In addition, recent studies in yeast *Saccharomyces cerevisiae* imply the presence of a pool of non-translating ribosomes under normal cellular conditions [47]. Several studies in eukaryotes have also demonstrated that under stress conditions the ribosomal subunits that are released through recycling, instead of engaging in new rounds of protein synthesis, associate to form a large pool of non-translating inactive ribosomes [48], [49]. In bacteria, the stress induced formation of hibernating ribosomes is also well documented [50]. The role of molecular chaperones like DnaK, Trigger factor, GroEL-GroES system in protecting cellular proteins under stress conditions is well established [51]. However, keeping in mind that the ribosome is present in large numbers in all living cells, the chaperoning ability of the ribosome might provide the cell with a cofactor independent and energetically inexpensive way for folding cellular proteins. Whether translationally inactive ribosomes present in the cells under stress conditions retain its chaperoning ability would therefore need to be investigated. However, the silencing of the chaperoning action of the ribosome in presence of P-site bound tRNA, as shown here, provides a way to segregate the peptidyl transferase and chaperoning activity of the ribosome thus enabling maintenance of its translational ability.

## Supporting Information

Figure S1 Effect of antibiotics, tRNA and heparin on BCAII reactivation. (**A**) Control experiments were performed to test the effect of tRNAs and antibiotics on BCAII reactivation in absence of chaperone. Bar diagram shows the effect of total *E. coli* tRNA (2), Met-tRNA (3), blasticidin (4), puromycin (5), erythromycin (6) and josamycin (7) on the self refolding of BCAII (1). The concentrations of all the tRNAs and antibiotics were the same at which their maximum inhibition of protein folding activity was observed with ribosome [Total *E. coli* tRNA = 10 µM, Met-tRNA = 5 µM, blasticidin = 10 µM, puromycin = 6 mM, erythromycin = 40 µM and josamycin = 360 µM]. (**B**) Comparison of BCAII reactivation after 30 minutes of refolding in absence of chaperone (1), in presence of 10 µM heparin (2), in presence of 70S ribosome (3), and in presence of 70S ribosome +10 µM heparin (4).
(TIF)

Figure S2 Effect of mutations of bDV RNA on CD spectra and its effect on the binding-release and reactivation of BCAII. (**A**) Wild type (wt) bDV RNA and its nine mutant variants were subjected to CD analysis to assess the secondary structure. The measurements were done in phosphate buffer (20 mM) using a quartz cuvette with a path length of 1 cm in a Jasco J-815 CD spectrometer with an RNA concentration of 0.1 µM. (**B**) Filter binding studies on the time course of interaction between BCAII and wt bDV RNA (•), delG2252bDV RNA (▪) are shown here. (**C**) Comparison of the BCAII reactivation after 30 minutes of refolding in absence of chaperone (1), and in presence of wt bDV RNA (2), delG2252bDV RNA (3), delG2252bDV RNA + wt RNA2 (4).
(TIF)

Table S1 Summary of buffer systems used in this study.
(DOC)

Table S2 Summary of dissociation constants of antibiotics.
(DOC)
