# Supplementary material for: Impact of P-Site tRNA and Antibiotics on Ribosome Mediated Protein Folding: Studies Using the Escherichia coli Ribosome
Source: PLoS One. 2014 Jul 7;9(7):e101293. doi: 10.1371/journal.pone.0101293 (PMC4085065; doi:10.1371/journal.pone.0101293)
Supplement: Table S1 — Summary of buffer systems used in this study. (DOC) [file pone.0101293.s003.doc]

Table S1: Summary of buffer systems used in this study.

| No | Buffer Name | Buffer Composition | Reference |
| --- | --- | --- | --- |
| 1 | Refolding Buffer (RB) | 50 mM Tris-HCl (pH 7.5), 10 mM MgCl_2_, 100 mM NaCl | [10], [13] |
| 2 | P-site binding buffer (Buffer-P) | 50 mM Tris-HCl (pH 7.5), 7 mM MgCl_2_, 30 mM KCl, 70 mM NH_4_Cl | [23] |
| 3 | A-site binding buffer (Buffer-A) | 50 mM Tris-HCl (pH 7.5), 20 mM MgCl_2_, 30 mM KCl, 70 mM NH_4_Cl | [23] |
| 4 | Tetracycline binding buffer (Tet- Buffer) | 50 mM Tris-HCl (pH 7.4), 5 mM Mg(OAc)_2_, 100 mM NH_4_Cl, 1 mM DTT * | [24] |
| 5 | Blasticidin binding buffer | 100 mM Tris-HCl (pH 7.2), 10 mM MgCl_2_ , 100 mM NH_4_Cl (pH 7.2), 6 mM β-Mercaptoethanol | [27] |
| 6 | Puromycin binding buffer | 50 mM Tris-HCl (pH 7.6), 10 mM MgCl_2_ , 50 mM KCl, 6 mM β-Mercaptoethanol | [25] |
| 7 | Erythromycin and josamycin binding buffer | 20 mM Tris-HCl (pH 7.6), 10 mM MgCl_2_,  150 mM NH_4_Cl, 6 mM β-Mercaptoethanol | [26] |

* 1 mM of DTT was added to this buffer in this study.
