# Supplementary material for: Impact of P-Site tRNA and Antibiotics on Ribosome Mediated Protein Folding: Studies Using the Escherichia coli Ribosome
Source: PLoS One. 2014 Jul 7;9(7):e101293. doi: 10.1371/journal.pone.0101293 (PMC4085065; doi:10.1371/journal.pone.0101293)
Supplement: Table S2 — Summary of dissociation constants of antibiotics. (DOC) [file pone.0101293.s004.doc]

Table S2: Summary of dissociation constants of antibiotics.

| No | Antibiotic | K_d_ | Reference |
| --- | --- | --- | --- |
| 1 | Blasticidin | 2 x 10^-6^ M | [35] |
| 2 | Erythromycin | 7.6-7.8 nM | [37] |
| 3 | Josamycin | 7.0- 9.3 nM | [37] |
